# Treatment Strategy for Dialysis Patient with Urothelial Carcinoma

**DOI:** 10.3390/diagnostics11111966

**Published:** 2021-10-22

**Authors:** Yun-Ching Huang, Yu-Liang Liu, Miao-Fen Chen, Chih-Shou Chen, Chun-Te Wu

**Affiliations:** 1 Division of Urology, Department of Surgery, Chang Gung Memorial Hospital at Chiayi, Chiayi 613, Taiwan; armsliu@cgmh.org.tw (Y.-L.L.); cv7589@cgmh.org.tw (C.-S.C.); 2 Department of Medicine, College of Medicine, Chang Gung University, Taoyuan 333, Taiwan; 3 Department of Radiation Oncology, Chang Gung Memorial Hospital at Chiayi, Chiayi 613, Taiwan; miaofen@cgmh.org.tw; 4 Department of Urology, Chang Gung Memorial Hospital at Keelung, Keelung 204, Taiwan

**Keywords:** urinary tract, bladder, neoplasm, nephroureterectomy, cystectomy, dialysis, urothelial carcinoma

## Abstract

To investigate postoperative complications and oncologic outcomes of prophylactic nephroureterectomy and/or cystectomy in dialysis patients with urothelial carcinoma (UC), we retrospectively reviewed the records of dialysis patients with UC and a final status of complete urinary tract extirpation (CUTE, i.e., the removal of both kidneys, ureters, and bladder) between January 2004 and December 2015. Patients undergoing dialysis after initial radical nephroureterectomy and/or cystectomy were excluded. Eighty-four and 27 dialysis patients, undergoing one-stage and multi-stage CUTE, were enrolled in this study, respectively. Demographic, medical, perioperative, and pathologic features were collected to determine variables associated with oncologic outcomes. Although there was no significant difference in mortality between the 2 groups (*p* = 0.333), all 5 (4.5%) patients with Clavien–Dindo grade 5 complications were from the one-stage CUTE group. On multivariate logistic regression analysis, advanced age (*p* = 0.042) and high Charlson comorbidity index (CCI) (*p* = 0.000) were related to postoperative major complications. Compared with multi-stage CUTE, one-stage CUTE had no overall, cancer-specific, and recurrence-free survival benefits (all *p* > 0.05). According to multivariate analysis with Cox regression, age > 70 years (HR 2.70, 95% CI 1.2–6.12; *p* = 0.017), CCI ≥ 5 (HR 2.16, 95% CI 1.01–4.63; *p* = 0.048), and bladder cancer stage ≥ 3 (HR 12.4, 95% CI 1.82–84.7; *p* = 0.010) were independent, unfavorable prognostic factors for the overall survival. One-stage CUTE is not associated with superior oncologic outcomes, and all perioperative mortalities in our series occurred in the one-stage CUTE group. Our data do not support prophylactic nephroureterectomy and/or cystectomy for uremic patients with UC.

## 1. Introduction

Patients with end-stage renal disease (ESRD), who are on dialysis, have an increased risk of developing urological cancers, including renal cell carcinoma (RCC) and urothelial carcinoma (UC) [1]. In Western countries, the predominant urinary tract malignancy in dialysis patients is RCC. However, UC is the most common malignancy in long-term dialysis patients in Taiwan, with a standardized incidence ratio (the ratio of observed to expected number of cancer cases) of 48.2 and an estimated incidence of almost 2%, after a mean dialysis duration of 46.5 months [2]. Although the reason for such a high incidence of UC among dialysis patients in Taiwan is still unknown, ingestion of Aristolochia-based herbal remedies [3], groundwater containing arsenic [4], analgesic abuse [5], immunosuppressive status [6], and chronic bladder irritation (decreased urinary wash effect) [7] have been suggested as potentially causal factors.

The role of one-stage complete urinary tract extirpation (CUTE, i.e., bilateral nephroureterectomy with cystectomy or cystoprostatectomy) in dialysis patients with UC remains controversial. Compared with non-dialysis patients, patients with UC on dialysis are more likely to have multifocal lesions throughout the urinary tract and a high recurrence rate [8]. Furthermore, early-stage synchronous and metachronous tumors may be difficult to identify using imaging. In view of a non- or poorly functional urinary tract that may have the potential for malignant transformation and to avoid repeat anesthesia, one-stage CUTE has been of interest as a therapeutic option in UC with ESRD [9,10]. In contrast, despite improvements in surgical techniques, anesthetic delivery, and perioperative care, the risk of post-surgical complications (including mortality) associated with ESRD argue against routine CUTE in dialysis patients with UC. Yossepowitch et al. reported that 2 of the 4 patients undergoing one-stage CUTE died soon after the operation and 1 had a Clavien–Dindo grade IV complication [11]. Sato et al. also found that bladder UC in dialysis patients can reportedly be treated using the same strategy as that for non-dialysis patients, and immediate cystectomy was performed only in patients with muscle-invasive bladder cancer or high-grade cT1 tumor [12].

The risks and benefits of prophylactic removal of benign, but non- or poorly functioning, segments of the upper and lower urinary tract at the time of UC remains unclear. However, owing to its relatively rare entity, few data exist on perioperative complications and oncologic outcomes in dialysis patients who have undergone one-stage versus multi-stage CUTE. The present study compares patients who have undergone one-stage versus multi-stage CUTE. We hypothesized that a one-stage CUTE procedure would have a high complication rate and better oncologic outcomes, compared with stepwise CUTE in multiple surgical procedures.

## 2. Materials and Methods

### 2.1. Study Population

After the study design was approved and the need for informed consent was waived by the institutional review board (IRB No. 202100779B0), we retrospectively reviewed dialysis patients with newly diagnosed UC, who underwent CUTE at our hospital from January 2004 to December 2015. At our institution, radical nephroureterectomy with bladder cuff excision is recommended in dialysis patients with upper urinary tract urothelial cell carcinoma (UTUC), regardless of tumor stage and location. Radical cystectomy was the standard treatment for dialysis patients with muscle-invasive or recurrent bladder cancer. To avoid any differences, in terms of pathologic details and complications, between the patients with and without CUTE, the inclusion criteria were dialysis patients with pathologically confirmed UC and a final status of CUTE. We excluded patients who were started on dialysis after initial radical surgery from the study cohort. Some of these patients underwent one-stage CUTE after being counseled about the benefits and adverse effects of CUTE by the treating urologist and anesthesiologist. Other patients underwent multi-stage CUTE for metachronous UC.

### 2.2. Pathological Examination

All the tumors were graded as low- and high-grade, according to the World Health Organization/International Society of Urologic Pathology, and staged using the 8th edition of the American Joint Committee on Cancer Staging Manual by urologic pathologists at our institution. The final pathologic features were determined according to the pathologic findings at the time of radical nephroureterectomy and/or cystectomy or cystoprostatectomy.

### 2.3. Postoperative Follow-Up

Although the follow-up schedules for our patients were slightly different, depending on our physicians, in general, the postoperative follow-up for dialysis patients with remnant kidneys and/or bladder after initial surgery involved cystoscopy with/without retrograde pyelogram at a 3-month interval for the first 2 years, 6-month interval for the subsequent 2 years, and then once every year. Cross-sectional imaging (abdominopelvic computerized tomography or magnetic resonance urography) and chest radiography were performed annually or when hematuria occurred during the follow-up period. Chest computerized tomography and bone scan were performed on demand in the selected patients.

### 2.4. Outcome Measures

To determine the impact of the therapeutic strategy on postoperative complications and survival, patients were analyzed by stratification into group 1 (all cases who received CUTE in 1 stage) and 2 (all cases who received CUTE in multiple stages).

Demographic, medical, perioperative, and pathologic features were collected for determining variables that affected outcomes. Demographic characteristics included gender, age, active smoking status, and body mass index (BMI). Medical details included the renal replacement therapy method, history of abdominal surgery, and Charlson comorbidity index (CCI). Perioperative characteristics included the American Society of Anesthesiologist (ASA) score, operative methods, and postoperative complications. Pathologic data included the tumor location, stage, grade, lymphovascular invasion, carcinoma in situ, and surgical margin.

Complication grades were determined using the Clavien–Dindo classification of surgical complications [13], which is a standardized and validated method, recommended by the International Consultation on Urological Diseases-European Association of Urology International Consultation on Bladder Cancer [14]. Complications occurring within the first 90 days after surgery or during the hospitalization, whichever was longer, were included in the study. Grade 3 to 5 complications were categorized as major complications [15,16,17].

Survival time was defined as the date of the first radical surgery until the most recent visit or death (cancer-specific or any other cause). Recurrence time was calculated from the date of the initial radical surgery to the time of first recurrence, including local recurrence in the tumor bed, lymph nodes, or distant metastasis. Metachronous UC was not included for the calculation of recurrence-free survival in the current patient population [18,19,20].

### 2.5. Statistical Analysis

All the data analysis was performed using SPSS version 20 (IBM Corp., Armonk, NY, USA). Descriptive statistics were reported using median values, with range for continuous variables and proportions for categorical variables. The differences in the continuous and categorical outcomes were evaluated using the two-tailed *t* test and Fisher’s exact test with the Chi-square test, respectively. Univariable and multivariable logistic regression were used for evaluating the odds ratios (OR) and predictive probability of major complications, including all the clinically meaningful covariates. Overall, cancer-specific, and recurrence-free survival curves were assessed using the Kaplan-Meier method, with the log-rank test. We used univariate analysis and multivariate analysis with Cox proportional hazards regression, in order to evaluate the potential predictive factors for overall, cancer-specific, and recurrence-free survival. Owing to sample size considerations, only variables that were identified with *p* < 0.05 by the univariate analysis were considered for further multivariate analysis. The hazard ratio (HR) was set at a 95% confidence interval (CI). A *p* value < 0.05 was considered statistically significant.

## 3. Results

### 3.1. Study Population

**Figure 1:**
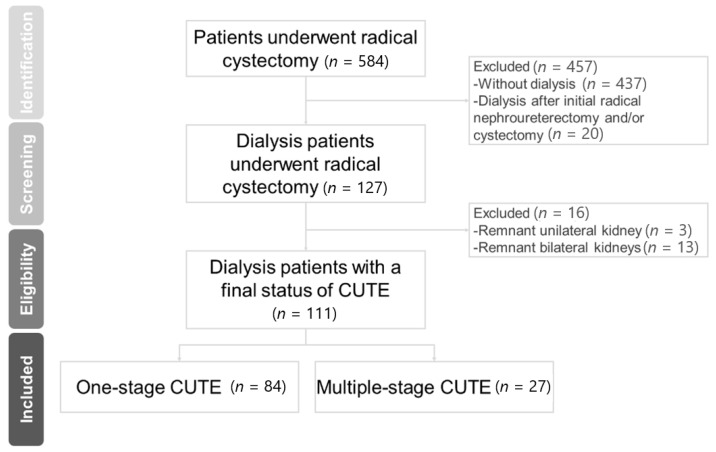
Patient enrollment flowchart. CUTE, complete urinary tract extirpation.

Overall, 111 dialysis patients with a final status of CUTE were identified for analysis (Figure 1). The median age was 62.0 years (range, 23.6–83.4 years). Female patients accounted for 62.2% of the study population (Table 1). 

The median length of follow-up after surgery, for the entire study cohort, was 73.4 months (range, 2 days to 194 months). The median time interval of dialysis before the first radical surgery was 5 years (range, 0.3–19.0 years). At the last follow up, 5 (4.5%) patients had died from perioperative complications, 6 (5.4%) died from cancer-related causes, 23 (20.7%) died from unrelated causes, and 13 (11.7%) had recurrent disease.

From this cohort, 84 (75.7%) patients received one-stage CUTE and 27 (24.3%) patients underwent multi-stage procedures, with a final status of CUTE. The median time for progression to the second and third radical surgery was 40.1 months (range 1.1–127.9 months) and 72.0 months (range, 24.3–119.2 months), respectively. There was no statistically significant difference in the gender, age, smoking, BMI, renal replacement therapy method, abdominal surgery history, CCI, ASA score, operative method, and adjuvant therapy between one-stage and multi-stage CUTE groups (all, *p* > 0.05).

### 3.2. Pathologic Features

Tumor locations at the time of CUTE were 14 (12.6%) unilateral UTUCs, 17 (15.3%) bladder UCs, 21 (18.9%) bilateral UTUCs, 19 (17.1%) unilateral UTUCs plus synchronous bladder UCs, and 33 (29.7%) bilateral UTUCs plus synchronous bladder UCs (Table 2). All 7 patients presented with no residual tumor, at the time of CUTE procedure were in the one-stage CUTE group and with a history of bladder cancer, previously undergoing transurethral resection (TUR). Interestingly, 24 (21.6%) patients had no UTUC at the time of CUTE, including 22 patients who received prophylactic nephroureterectomy in the one-stage CUTE group and 2 in the multi-stage CUTE group. Of the 2 patients who had bladder cancer with no UTUC, 1 prepared for one-stage CUTE but suffered from intraoperative hypotension during bilateral nephroureterectomy and, therefore, received cystectomy in the second surgical procedure; the remaining 1, previously treated with cystectomy, received bilateral nephroureterectomy because of bilateral severe hydronephrosis. 

There were 42 patients with no residual bladder cancer at the time of CUTE, of whom 31 had UTUC plus synchronous bladder UC previously treated with TUR, and 11 of whom had UTUC previously treated with prophylactic cystectomy. Four (3.6%) patients, without preoperatively detectable RCC, had a confirmed pathologic diagnosis. Among the male patients, 7 (16.7%) had incidental prostate adenocarcinoma.

The pathological stage of the CUTE specimen was locally advanced UTUC in 13 (11.7%) patients and metastatic UTUC in 9 (8.1%) patients. Muscle-invasive bladder cancer was diagnosed in 17 (15.3%) patients and metastatic bladder cancer in 4 (3.6%), at the time of CUTE.

There was no significant difference in the tumor location, UTUC highest stage, bladder stage, tumor grade, lymphovascular invasion, carcinoma in situ, and positive surgical margin between the patients who underwent one-stage or multi-stage CUTE.

### 3.3. Perioperative Complications

Overall, 94 (84.7%) patients experienced at least one complication, of whom 54 (48.6%) patients had minor complications, and 40 (36.0%) patients had major complications (Table 3). Thirty (27.0%) patients developed Clavien–Dindo grade 3 complications during the intra-operative and post-operative period, with the most common type of complication being arteriovenous shunt dysfunction (10.8%). There were no intraoperative deaths. All 5 (4.5%) patients who developed Clvien–Dindo grade 5 complication (death within 90 days of surgery) were in the one-stage CUTE group. Causes of death included cardiac arrest (*n* = 2), pancreatic injury (*n* = 1), intra-abdominal abscess (*n* = 1), and acute respiratory distress syndrome (*n* = 1). There was no statistically significant difference in the incidence of major complications between the one-stage and multi-stage CUTE groups.

### 3.4. Predictive Probability of Major Complication and Mortality

According to the univariate and multivariate logistic regression analysis, those aged >70 years (OR 3.72, 95% CI 1.34–10.4; *p* = 0.012) and CCI ≥5 (OR 4.78, 95% CI 1.98–11.5; *p* = 0.000) were identified for the prediction of subsequent major complications (Table 4). 

In univariate and multivariate logistic regression models, there was no independent factor associated with mortality.

### 3.5. Survival and Recurrence

**Figure 2:**
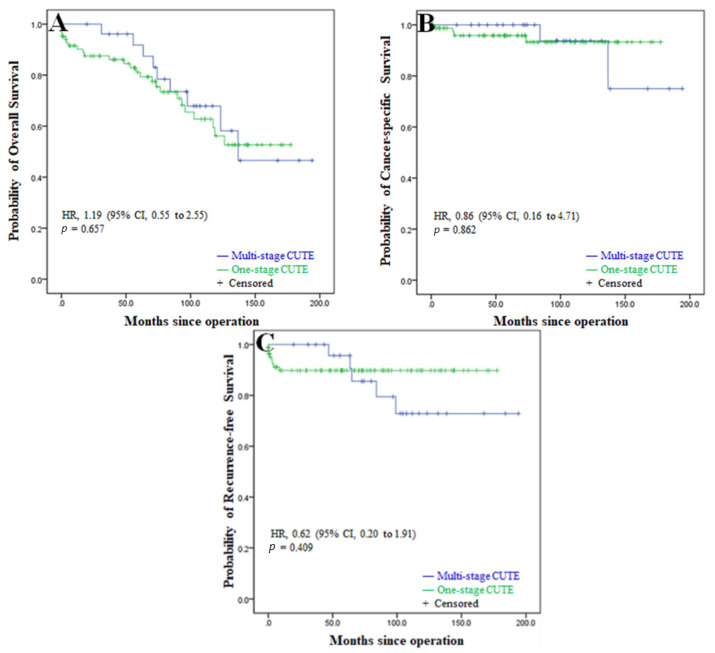
Kaplan-Meier estimates of (**A**) overall survival, (**B**) cancer-specific survival, and (**C**) recurrence-free survival in dialysis patients with urothelial carcinoma undergoing one-stage CUTE versus multi-stage CUTE. HR, hazard ratio; CI, confidence interval; CUTE, complete urinary tract extirpation.

The median survival in the one-stage and multi-stage CUTE were 70.4 months (range, 3 days to 177.5 months) and 97.0 months (19.7 to 194.1 months), respectively. There was no statistically significant difference in the 5-year overall (79.9% vs. 89.3%, *p* = 0.657), cancer-specific (92.7% vs. 93.8%, *p* = 0.862) and recurrence-free (89.8% vs. 91.7%, *p* = 0.409) survival between the one-stage and multi-stage CUTE groups (Figure 2).

According to multivariate analysis, with Cox proportional hazards regression, those aged > 70 years (HR 2.70, 95% CI 1.2–6.12; *p* = 0.017), CCI ≥ 5 (HR 2.16, 95% CI 1.01–4.63; *p* = 0.048), and bladder cancer stage ≥ 3 (HR 12.4, 95% CI 1.82–84.7; *p* = 0.010) were independent, unfavorable prognostic factors for the overall survival (Table 5). In addition, UTUC highest stage ≥ 3 (HR 5.80, 95% CI 1.42–23.6; *p* = 0.014) and bladder cancer stage ≥ 3 (HR 57.3, 95% CI 5.42–605; *p* = 0.001) were independent, unfavorable prognostic factors for recurrence-free survival. 

## 4. Discussion

To date, whether one-stage CUTE is an optimal choice for dialysis patients with UC it is still a controversial issue. One-stage CUTE has been routinely recommended for patients with UC in Chinese-speaking countries, in part, due to the high incidence of synchronous and metachronous tumors [9,10,21,22,23]. Holton et al. advocated that one-stage CUTE can be performed effectively and safely in patients with diverse genitourinary pathologic characteristics [24]. Conversely, Kang et al. reported that there was no survival benefit between one- and two-step bilateral nephroureterectomy, and prophylactic cystectomy had a higher mortality rate [25]. Sato et al. came to a similar conclusion that patients with bladder UC should be managed without regard to ESRD status [12]. Although there is no consensus on the role of one-stage CUTE for dialysis patients with UC, our results suggest that prophylactic one-stage CUTE is not associated with significant benefits to overall, cancer-specific, and recurrence-free survival, compared to staged CUTE. Subsequent nephroureterectomy and/or cystectomy does not comprise the outcomes of patients with metachronous upper tract or lower tract UC. Moreover, prophylactic cystectomy complicates the possibility of kidney transplantation in young dialysis patients with UC, who are >2 years cancer-free after surgery.

Another reason for not recommending prophylactic nephroureterectomy and/or cystectomy is the risk of perioperative complications [26]. Although there was no significant difference in major complications between one-stage and multi-stage CUTE, all the patients with Clavien–Dindo grade 5 complications belonged to the one-stage CUTE group. The etiology of this higher rate of death is not entirely clear, but given the nature of deaths in the one-stage CUTE group, it is likely that the operation was a risk factor. Further study on the specific predictive factors is needed for clarifying this issue.

A unique finding is that women constitute a larger proportion of the total number of patients with UC and ESRD; this differs from Western countries, in which UC is more common in men [27,28]. Factors that may contribute to the development of UC in Taiwan include herbal remedies (aristolochic acid) [29], and women patients ingested more aristolochia-containing Chinese herbal medicines [30]. Furthermore, the dialysis population in Taiwan is skewed towards female predominance (ratio of 1.2:1) [31].

Bladder cancer tends to be pathologically more advanced in dialysis patients with a poor prognosis [11,12]. However, we found that most patients who underwent radical surgery were in an early stage. In our cohort, 80.1% of UTUC patients had localized disease (≤stage 2), and 80.9% of bladder UC patients had non-muscle invasive disease (≤stage 1). There are several possible explanations for the different results between the current and previous collaborative studies. Because of the high incidence of UC in dialysis patients in Taiwan, patients on dialysis were under rigorous medical surveillance by the hemodialysis center and were asked to visit clinicians, in the setting of urethral bloody discharge or gross hematuria. Patients on dialysis have higher risks for developing multifocal lesions throughout the urinary tract, with a high recurrence rate [8]. Therefore, early aggressive surgical intervention is recommended in dialysis patients with invasive or recurrent UC in Taiwan [9,10]. Furthermore, pathologic features were determined according to the time of radical nephroureterectomy and cystectomy.

Although the standardized and validated methodology for reporting adverse events exists, it is not routinely used for reporting the surgical complications in the urologic oncology, making it difficult to reliably compare the outcomes among different surgical techniques and surgeons’ competency [32]. The current study has rigorously presented the complications, according to the Clavien–Dindo system, in dialysis patients with UC. We found that the overall major complication and mortality rates were 36.0% and 4.5% in our cohort, respectively. Based on our study, advanced age and CCI ≥ 5 were at a higher risk of major surgical complications. Our rate is significantly higher than that of a large series of 1142 patients undergoing radical cystectomy, as reported by Shabsigh et al., with major complication and mortality rates of 13.4% and 1.7%, respectively [33]. The higher incidence of major complications is not surprising, as CUTE is a more invasive procedure than radical cystectomy; dialysis patients also have a higher baseline prevalence of comorbid conditions [34], electrolyte imbalance, and narrow therapeutic interval of fluid infusion [25]. Higher mortality rates were reported in one-stage CUTE (6%), but there was no mortality in the patients receiving multi-stage CUTE. Owing to the higher mortality rates for patients receiving one-stage CUTE, preoperative evaluation, specialty consultation, and appropriate risk mitigation strategies (including deferring one-stage CUTE) are important before proceeding to the surgery. 

Although certain cancers are more common among dialysis patients, malignancy is a relatively rare cause of death among uremic patients [35]. In our study, at the end of the follow-up, only 5.4% of the patients died from cancer-related causes, compared with 20.7% of the patients who died from unrelated causes. Independent predictors of mortality in dialysis patients include comorbid conditions, underlying renal disease process, age, country, race, psychosocial factors, poor nutrition, high salt intake, and residual kidney function [36]. Our multivariate analysis showed that older age and high CCI are unfavorable prognostic factors. These findings are consistent with those of Noh et al., who reported that older age (≥70.5 years) and high CCI (>4) were independently related to a higher risk of death in dialysis patients, and the HR for death was 4.6 times higher than that for the overall study population [37].

Many prognostic factors have been determined and can be used to predict the risk of disease recurrence and/or progression in patients with bladder cancer. Our multivariate analysis found that bladder cancer stages ≥3 were independent, unfavorable prognostic factors for overall and recurrence-free survival. These findings are similar to the guidelines of the American urological association and European association of urology, in which the most important prognostic factors after radical cystectomy are tumor stage [38,39]. In recent years, the lymphocyte-to-monocyte ratio has emerged as a novel prognostic biomarker, in both non-muscle-invasive bladder cancer patients receiving immunotherapy and muscle-invasive bladder cancer patients undergoing radical cystectomy [40]. Additional studies to strengthen the lymphocyte-to-monocyte ratio, as a predictor of progression, are warranted.

There are some limitations in our study. First, it is not a prospective, randomized control trial. Missing data (i.e., BMI) and selection bias (i.e., treatment choice) are inevitable. Second, the number of patients was too small to make definite conclusions, especially in the multi-stage CUTE group. In addition, some clinical characteristics (age, BMI, and CCI) in the multi-stage CUTE group varied between the initial radical surgery and subsequent removal of retained genitourinary tract. To compare the predictive factors of major complications and potential prognostic factors for survival, clinical features were documented during the initial radical surgery. Finally, patients who received medical or less aggressive surgical treatment, without a final status of CUTE, were not recruited; therefore, the results are not generally applicable to all dialysis patients with UC. Although definite recommendations cannot be made based on our relatively small sample size, to our knowledge, this is the first study to compare surgical strategies based on complications and survival in dialysis patients with UC. A large-scale and multi-center study is required for determining the clinical outcomes and optimal therapeutic strategy in uremic patients with UC.

## 5. Conclusions

Dialysis patients with UC undergoing CUTE have a high incidence of developing perioperative complications. There was no statistically significant difference in the major complications between one-stage and multi-stage CUTE; advanced age and high CCI were related to postoperative major complications. However, one-stage CUTE had no survival benefits, compared to multi-stage CUTE, and all peri-operative mortalities in our series occurred in the one-stage CUTE group. Therefore, prophylactic nephroureterectomy and/or cystectomy should not be routinely performed for uremic patients with UC. Old age, high CCI, and advanced bladder cancer stage are associated with decreased overall survival. Prospective, large-scale studies are needed for clarifying the optimal surgical strategy for dialysis patients with UC. 

## Figures and Tables

**Table 1:** Characteristics of dialysis patients undergoing CUTE.

Features	Total (*n* = 111)	One-Stage CUTE (*n* = 84)	Multiple-Stage CUTE (*n* = 27)	*p* Value
Gender Female, *n* (%) Male, *n* (%)	69 (62.2) 42 (37.8)	52 (61.9) 32 (38.1)	17 (63.0) 10 (37.0)	1.000
Age, years, median (range)	62.0 (23.6–83.4)	62.0 (23.6–83.4)	60.6 (28.0–75.1)	0.315
Current smoking, *n* (%)	9 (8.1)	7 (8.3)	2 (7.4)	1.000
BMI, kg/m^2^, median (range)	22.5 (16.2–38.1)	22.4 (16.2–38.1)	23.6 (17.6–33.2)	0.233
Renal replacement therapy ^a^ Peritoneal dialysis, *n* (%) Hemodialysis, *n* (%)	12 (11.4) 93 (88.6)	8 (10.1) 71 (89.9)	4 (15.4) 22 (84.6)	0.486
Abdominal surgery history, *n* (%)	36 (32.4)	24 (28.6)	12 (44.4)	0.157
CCI, median (range) ≤4, *n* (%) ≥5, *n* (%)	4 (3–9) 57 (51.4) 54 (48.6)	4 (3–9) 43 (51.2) 41 (48.8)	4 (4–9) 14 (51.9) 13 (48.1)	0.750 1.000
ASA score 2, *n* (%) 3, *n* (%) Unknown, *n* (%)	8 (7.2) 99 (89.2) 4 (3.6)	4 (4.8) 80 (95.2) 0 (0)	4 (14.8) 19 (70.4) 4 (14.8)	0.063
Operative methods Laparoscopic, *n* (%) Open, *n* (%)	36 (32.4) 75 (67.6)	28 (33.3) 56 (66.7)	8 (29.6) 19 (70.4)	0.816
Adjuvant therapy, *n* (%) ^b^	17 (15.3)	11 (13.1)	6 (22.2)	0.355

^a^ Six patients with kidney transplantation were excluded; ^b^ adjuvant therapy chemotherapy and radiotherapy were included; abbreviations: CUTE: complete urinary tract extirpation; BMI: body mass index; ASA: American Society of Anesthesiologists; CCI: Charlson comorbidity index.

**Table 2:** Pathological characteristics.

Tumor Location and Stage	Total (*n* = 111)	One-Stage CUTE (*n* = 84)	Multiple-Stage CUTE (*n* = 27)	*p* Value
Tumor location ^a^ Unilateral upper urinary tract Bladder Bilateral upper urinary tract Unilateral upper urinary tract + bladder Bilateral upper urinary tract + bladder No residual tumor ^b^	14 (12.6) 17 (15.3) 21 (18.9) 19 (17.1) 33 (29.7) 7 (6.3)	11 (13.1) 15 (17.9) 14 (16.7) 16 (19.0) 21 (25.0) 7 (8.3)	3 (11.1) 2 (7.4) 7 (25.9) 3 (11.1) 12 (44.4) 0 (0)	0.154
Upper urinary tract highest stage ^a^ 0a/0is I II III IV No tumor	30 (27.0) 22 (19.8) 13 (11.7) 13 (11.7) 9 (8.1) 24 (21.6)	23 (27.4) 17 (20.2) 8 (9.5) 8 (9.5) 6 (7.1) 22 (26.2)	7 (25.9) 5 (18.5) 5 (18.5) 5 (18.5) 3 (11.1) 2 (7.4)	0.252
Bladder stage ^a^ 0a/0is I II III IV No residual tumor	19 (17.1) 29 (26.1) 10 (9.0) 7 (6.3) 4 (3.6) 42 (37.8)	15 (17.9) 23 (27.4) 6 (7.1) 5 (6.0) 3 (3.6) 32 (38.1)	4 (14.8) 6 (22.2) 4 (14.8) 2 (7.4) 1 (3.7) 10 (37.0)	0.885
Tumor grade ^b^ Low High	5 (4.8) 99 (95.2)	4 (5.2) 73 (94.8)	1 (3.7) 26 (96.3)	1.000
Lymphovascular invasion	14 (12.6)	9 (10.7)	5 (18.5)	0.322
Carcinoma in situ	20 (18.0)	14 (16.7)	6 (22.2)	0.568
Positive surgical margin	5 (4.5)	3 (3.6)	2 (7.4)	0.594

Data are expressed as *n* (%); ^a^ tumor location and stage were determined according to the pathologic findings in the radical nephroureterectomy and cystectomy procedure; ^b^ seven patients with no residual tumor in the radical nephroureterectomy and cystectomy procedure were excluded; abbreviations: CUTE: complete urinary tract extirpation.

**Table 3:** Clavien–Dindo grade 0–5 complications after CUTE.

Grading	Total (*n* = 111)	One-Stage CUTE (*n* = 84)	Multiple-Stage CUTE (*n* = 27)	*p* Value
Grade 0	17 (15.3)	15 (17.9)	2 (7.4)	0.235
Grade 1	18 (16.2)	17 (20.2)	1 (3.7)	0.068
Grade 2	36 (32.4)	22 (26.2)	14 (51.9)	**0.018**
Grade 3 Surgical intervention Arteriovenous shunt dysfunction Wound dehiscence Rectovaginal or vaginal fistula Spleen laceration or splenectomy Rectal perforation Vaginal bleeding Enterolysis Intra-abdominal abscess Radiological intervention Endoscopic intervention	30 (27.0) 23 (20.7) 12 (10.8) 3 (2.7) 2 (1.8) 2 (1.8) 1 (0.9) 1 (0.9) 1 (0.9) 1 (0.9) 5 (4.5) 2 (1.8)	21 (25.0) 17 (16.7) 9 (10.7) 1 (1.2) 2 (2.4) 2 (2.4) 1 (1.2) 1 (1.2) 1 (1.2) 3 (3.6) 1 (1.2)	9 (33.3) 6 (22.2) 3 (11.1) 2 (7.4) 1 (3.7) 2 (7.4) 1 (3.7)	0.457
Grade 4, life-threatening organ dysfunction	5 (4.5)	4 (4.8)	1 (3.7)	1.000
Grade 5, death	5 (4.5)	5 (6.0)	0 (0)	0.333

Data are expressed as *n* (%); significant values are shown in bold; abbreviations: CUTE: complete urinary tract extirpation.

**Table 4:** Univariate and multivariate logistic regression for predictive factors of postoperative major complications and mortality.

	Major Complication				Mortality			
	Univariate		Multivariate		Univariate		Multivariate	
	OR (95% CI)	*p* Value	OR (95% CI)	*p* Value	OR (95% CI)	*p* Value	OR (95% CI)	*p* Value
Female gender (referent: male)	1.43 (0.64–3.24)	0.385			0.39 (0.06–2.43)	0.311		
Age (referent: <60 years) 60–70 >70	1.46 (0.55–3.92) 3.82 (1.47–9.97)	**0.021** (0.451) **(0.006)**	1.73 (0.60–4.99) 3.72 (1.34–10.4)	**0.042** (0.308) **(0.012)**	10,000 (0–10,000) 10,000 (0–10,000)	0.429 (0.997) (0.997)		
BMI ≥ 25 kg/m^2^ (referent: <25 kg/m^2^)	1.53 (0.40–4.63)	0.365			0.99 (0.10–9.92)	0.991		
Renal replacement therapy (referent: hemodialysis)	1.36 (0.17–2.26)	0.621			0 (0–10,000)	0.999		
Prior abdominal surgery (referent: absent)	1.20 (0.53–2.73)	0.665			0 (0–10,000)	0.998		
CCI (referent: ≤4)	4.85 (2.08–11.3)	**0.000**	4.78 (1.98–11.5)	**0.000**	4.48 (0.49–41.4)	0.186		
ASA score 3 (referent: ASA 2)	1.79 (0.34–9.33)	0.489			10,000 (0–10,000)	0.999		
Operative methods (referent: laparoscopy)	2.11 (0.87–5.11)	0.097			1.97 (0.21–18.3)	0.550		

Major complications were defined as Clavien–Dindo grades 3 to 5 complications; significant values are shown in bold; abbreviations: OR: odds ratio; CI confidence: interval; BMI: body mass index; ASA: American Society of Anesthesiologists; CCI: Charlson comorbidity index.

**Table 5:** Univariate and multivariate analysis of potential prognostic factors for overall, cancer-specific, and recurrence-free survival in the dialysis patients undergoing CUTE.

	Overall Survival				Cancer-Specific Survival				Recurrence-Free Survival			
	Univariate		Multivariate		Univariate		Multivariate		Univariate		Multivariate	
Variables	HR (95%CI)	*p* Value	HR (95%CI)	*p* Value	HR (95%CI)	*p* Value	HR (95%CI)	*p* Value	HR (95%CI)	*p* Value	HR (95%CI)	*p* Value
Female gender (referent: male)	0.52 (0.27–1.03)	0.061			0.42 (0.08–2.13)	0.296			0.32 (0.10–0.98)	**0.046**	0.40 (0.10–1.59)	0.191
Age (referent: < 60 years) 60–70 >70	1.05 (0.40–2.78) 3.87 (1.79–8.36)	**0.001** (0.922) **(0.001)**	1.1 (0.41–2.95) 2.70 (1.20–6.12)	**0.043** (0.855) **(0.017)**	0 (0–1000) 1.87 (0.31–11.3)	0.793 (0.973) (0.496)			0 (0–10,000) 1.53 (0.50–4.71)	0.761 (0.959) (0.461)		
Current smoking (referent: absent)	0.49 (0.12–2.04)	0.324			0.04 (0–6701)	0.605			0.77 (0.10–5.94)	0.803		
BMI ≥ 25 kg/m^2^ (referent: <25 kg/m^2^)	0.75 (0.31–1.84)	0.528			0.03 (0–388)	0.470			1.41 (0.35–5.62)	0.631		
Renal replacement therapy (referent: hemodialysis)	0.69 (0.21–2.26)	0.535			0.04 (0–1717)	0.551			0.76 (0.10–6.02)	0.797		
Prior abdominal surgery (referent: absent)	0.62 (0.30–1.31)	0.212			0.26 (0.03–2.30)	0.226			0.51 (0.14–1.86)	0.309		
CCI (referent: ≤4)	2.51 (1.23–5.12)	**0.012**	2.16 (1.01–4.63)	**0.048**	1.41 (0.27–7.31)	0.680			1.88 (0.61–5.75)	0.269		
ASA score 3 (referent: ASA 2)	1.44 (0.34–6.07)	0.616			23.1 (0–10,000)	0.651			22.9 (0–10,000)	0.539		
Operative methods (referent: laparoscopic)	1.03 (0.50–2.11)	0.942			0.89 (0.16–4.86)	0.888			1.16 (0.36–3.77)	0.804		
UTUC stage 3/4 (referent: ≤stage 2)	1.68 (0.78–3.61)	0.183			11.0 (1.92–63.0)	**0.007**	1000 (0–10,000)	0.943	4.03 (1.35–12.0)	**0.012**	5.80 (1.42–23.6)	**0.014**
Bladder stage 3/4 (referent: ≤stage 2)	6.61 (2.76–15.8)	**0.000**	12.4 (1.82–84.7)	**0.010**	34.0 (5.41–213)	**0.000**	1000 (0–10,000)	0.931	24.3 (7.89–74.7)	**0.000**	57.3 (5.42–605)	**0.001**
Tumor grade (referent: low grade)	0.63 (0.15–2.66)	0.526			21.7 (0–10,000)	0.737			0.62 (0.08–4.79)	0.649		
Lymphovascular invasion (referent: absent)	2.54 (1.04–6.18)	**0.040**	0.30 (0.04–2.18)	0.235	5.70 (1.03–31.6)	**0.046**	0 (0–10,000)	0.943	9.13 (3.0327.5)	**0.000**	0.25 (0.02–2.91)	0.270
Positive surgical margin (referent: absent)	2.68 (0.62–11.6)	0.186			0.05 (0–10,000)	0.822			9.15 (2.42–34.6)	**0.001**	3.54 (0.56–22.3)	0.178
Carcinoma in situ (referent: absent)	0.86 (0.36–2.09)	0.741			0.03 (0–154.3)	0.427			0.04 (0–15.1)	0.281		
Adjuvant therapy (referent: absent)	1.77 (0.77–4.07)	0.181			7.19 (1.44–36.0)	**0.016**	1.30 (0.15–11.1)	0.812	5.57 (1.87–16.6)	**0.002**	0.56 (0.12–2.67)	0.465
Clavien surgical complications (referent: ≤2)	3.01 (1.51–6.0)	**0.002**	2.06 (1.0–4.24)	0.051	5.10 (0.89–29.3)	0.067			3.55 (1.16–10.9)	**0.026**	3.29 (0.72–15.1)	0.126
One-stage CUTE (referent: multiple-stage)	1.19 (0.55–2.55)	0.657			0.86 (0.16–4.71)	0.862			0.62 (0.20–1.91)	0.409		

Significant values are shown in bold; abbreviations: CUTE: complete urinary tract extirpation; HR: hazard ratio; CI: confidence interval; BMI: body mass index; ASA: American Society of Anesthesiologists; CCI: Charlson comorbidity index; UTUC: upper urinary tract urothelial carcinoma.

## Data Availability

Data availability is limited, due to institutional data protection law and confidentiality of patient data.
